# Interferon-λ1 Linked to a Stabilized Dimer of Fab Potently Enhances both Antitumor and Antiviral Activities in Targeted Cells

**DOI:** 10.1371/journal.pone.0063940

**Published:** 2013-05-16

**Authors:** Donglin Liu, Chien-Hsing Chang, Edmund A. Rossi, Thomas M. Cardillo, David M. Goldenberg

**Affiliations:** 1 IBC Pharmaceuticals, Inc., Morris Plains, New Jersey, United States of America; 2 Immunomedics, Inc., Morris Plains, New Jersey, United States of America; 3 Garden State Cancer Center, Center for Molecular Medicine and Immunology, Morris Plains, New Jersey, United States of America; McMaster University, Canada

## Abstract

The type III interferons (IFNs), comprising IFN-λ1, IFN-λ2, and IFN-λ3, behave similarly to IFN-α in eliciting antiviral, antitumor, and immune-modulating activities. Due to their more restricted cellular targets, IFN-λs are attractive as potential alternatives to existing therapeutic regimens based on IFN-αs. We have applied the DOCK-AND-LOCK™ method to improve the anti-proliferative potency of IFN-λ1 up to 1,000-fold in targeted cancer cell lines by tethering stabilized Fab dimers, derived from hRS7 (humanized anti-Trop-2), hMN-15 (humanized anti-CEACAM6), hL243 (humanized anti-HLA-DR), and c225 (chimeric anti-EGFR), to IFN-λ1 site-specifically, resulting in novel immunocytokines designated (E1)-λ1, (15)-λ1, (C2)-λ1, and (c225)-λ1, respectively. Targeted delivery of IFN-λ1 via (15)-λ1 or (c225)-λ1 to respective antigen-expressing cells also significantly increased antiviral activity when compared with non-targeting (C2)-λ1, as demonstrated in human lung adenocarcinoma cell line A549 by (15)-λ1 against encephalomyocarditis virus (EC_50_ = 22.2 pM versus 223 pM), and in human hepatocarcinoma cell line Huh-7 by (c225)-λ1 against hepatitis C virus (EC_50_ = 0.56 pM versus 91.2 pM). These promising results, which are attributed to better localization and stronger binding of IFN-λ1 to antibody-targeted cells, together with the favorable pharmacokinetic profile of (E1)-λ1 in mice (T_1/2_ = 8.6 h), support further investigation of selective prototypes as potential antiviral and antitumor therapeutic agents.

## Introduction

Interferons (IFNs) are class II cytokines with antitumor and antiviral effects [1], and have been explored extensively as therapeutic agents in a variety of diseases [2]–[4]. However, their clinical usefulness to date, as exemplified by IFN-α, IFN-β, and IFN-γ, is limited by a short circulating half-life, systemic toxicity, and suboptimal responses in patients [5]. The discovery of the IFN-λ family in 2003 introduced a new opportunity to develop alternative IFN agents [6], [7].

IFN-λs are type III interferons, comprising IFN-λ1, IFN-λ2 and IFN-λ3 (also referred to as interleukin-29, 28A, and 28B, respectively), each encoded by independent genes on chromosome 19 [6], [7]. IFN-λ2 and IFN-λ3 are highly homologous, with 96% amino acid identity, and IFN-λ1 shares approximately 81% homology [7]. IFN-λs, like the type I IFNs, which comprise both IFN-α and IFN-β, trigger signal transduction via the JAK/STAT pathway, including the activation of JAK1 and TYK2 kinases, the phosphorylation of STAT proteins, and the activation of the transcription complex of IFN-stimulated gene factor 3 [8], [9].

A major difference between type III and type I IFNs is the distribution of their respective receptor complexes. IFN-α/β signals through two widely expressed type I interferon receptors, which is at least partially responsible for the systemic toxicity associated with IFN-α/β therapy [10]. In contrast, IFN-λs signal through a heterodimeric receptor complex comprising the IFN-λ receptor 1 (IFN-λR1) and the IL-10 receptor 2 (IL-10R2). Whereas IL-10R2 is ubiquitously expressed among hematopoietic and nonhematopoietic cells, IFN-λR1 has a more restricted expression pattern, with the highest levels in epithelial cells, melanocytes, and hepatocytes, and the lowest level in primary central nervous system cells [11]. Although blood immune cells express IFN-λR1, they exhibit impaired response to IFN-λs due to the secretion of a short spliced variant of IFN-λR1 that inhibits the effect of IFN-λ1 [12]. The limited responsiveness of neuronal cells and immune cells also contributes to the reduced toxicity of IFN-λs, compared to type I IFN [8], [12].

IFN-λs display structural features similar to IL-10-related cytokines, but exhibit type I IFN-like antiviral and anti-proliferative activity [8], [13], [14]. For example, studies have demonstrated that IFN-λ1 and IFN-λ2 can reduce viral replication or the cytopathic effect of various viruses, including DNA viruses, such as hepatitis B virus [14], [15] and herpes simplex virus 2 [16]; positive-sense, single-stranded RNA viruses, such as encephalomyocarditis virus (EMCV) [7] and hepatitis C virus (HCV) [14], [15], [17], [18]; negative-sense, single-stranded RNA viruses, such as vesicular stomatitis virus [6], [18] and influenza-A virus [19]; and double-stranded RNA viruses, such as rotavirus [20]. IFN-λ3 was identified from genetic studies as a key cytokine in HCV infection [21]–[23], and has the most potent activity against EMCV [24]. The anti-proliferative activity of IFN-λs has also been documented in several human cancer cell lines, including neuroendocrine carcinoma BON1 [25], glioblastoma LN319 [26], immortalized keratinocyte HaCaT [27], melanoma F01 [28], and esophageal carcinoma TE-11 [29]. In animal models, IFN-λs induce tumor apoptosis and elimination through both innate and adaptive immune responses, suggesting that local delivery of IFN-λ might be a useful strategy for the treatment of human malignancies [30], [31].

Human IFN-λ1 conjugated to a 20-kDa polyethylene glycol (PEG-IFN-λ1) is currently under clinical development for the treatment of chronic HCV infection. In a phase Ib study, antiviral activity was observed at each dose (0.5–3.0 µg/kg), with viral load reduced 2.3 to 4.0 logs when PEG-IFN-λ1 was administered to genotype 1 HCV patients who relapsed after IFN-α therapy [32], [33]. A phase IIb study [34] reported that HCV patients (genotypes 1 and 4) had significantly higher response rates to treatment with PEG-IFN-λ1 versus PEG-interferon α-2a, with the additional advantages of PEG-IFN-λ1 over PEG-interferon α-2a including lower frequency of adverse events, decreased frequency of flu-like symptoms, anemia, and musculoskeletal symptoms, and rarely observed neutropenia and thrombocytopenia. However, higher rates of hepatotoxicity were seen with the highest-dose PEG-IFN-λ1 compared with PEG-interferon α-2a [34].

The DOCK-AND-LOCK™ (DNL™) platform technology [35]–[37] is a powerful method to construct novel agents of defined composition and retained bioactivity by site-specific conjugation of two types of modules, one containing the dimerization and docking domain (DDD) of cAMP-dependent protein kinase A (PKA), referred to as the DDD module, and the other bearing the anchoring domain (AD) of an interactive A-kinase anchoring protein (AKAP), referred to as the AD module. Among the distinctive features of DNL are the spontaneous formation of a dimer of the DDD module and the self-assembly of the DDD module with the AD module into a non-covalent complex, which is subsequently rendered covalent with disulfide bonds to enhance stability *in vivo* [36]. The amino acid sequences of a pair of DDD and AD linkers useful for the DNL conjugation are termed DDD2 and AD2, respectively. We have previously demonstrated in preclinical studies the superiority of antibody-targeted delivery of IFN-α-2b using novel conjugates, made by the DNL method, comprising four molecules of IFN-α-2b covalently linked to a humanized IgG with specificity for CD20 [37] or HLA-DR [38]. Here, we report the development of another class of DNL conjugates, referred to as 2(Fab)-λ1 for targeted delivery of IFN-λ1 via a stabilized dimer of Fab derived from a humanized or chimeric antibody, and show that the greatly enhanced antitumor as well as antiviral potency observed for the four prototypes can be attributed to their increased localization and stronger binding to target cells.

## Materials and Methods

### Antibodies and Reagents

Humanized antibodies, including veltuzumab (anti-CD20), hRS7 (anti-Trop2), hMN-15 (anti-CEACAM6), hL243 (anti-HLA-DR), and h225 (anti-EGFR), and control modules, such as hRS7-Fab-AD2 and hMN-15-IgG-AD2, were provided by Immunomedics, Inc. Recombinant human IFN-λ1(rhIFN-λ1), mouse anti-human IFN-λ1, and FITC mouse anti-human HLA-ABC were purchased from R&D Systems (Minneapolis, MN). Polyclonal rabbit antibodies for STAT1, STAT3, pY-STAT1, and pY-STAT3 were obtained from Cell Signaling Technology (Danvers, MA). Polyclonal rabbit anti-STAT2 and anti-pY-STAT2 antibodies were from Santa Cruz Biotechnology (Santa Cruz, CA) and Millipore Corporation (Billerica, MA), respectively.

### Expression and Purification of DNL Modules

**Figure 1:**
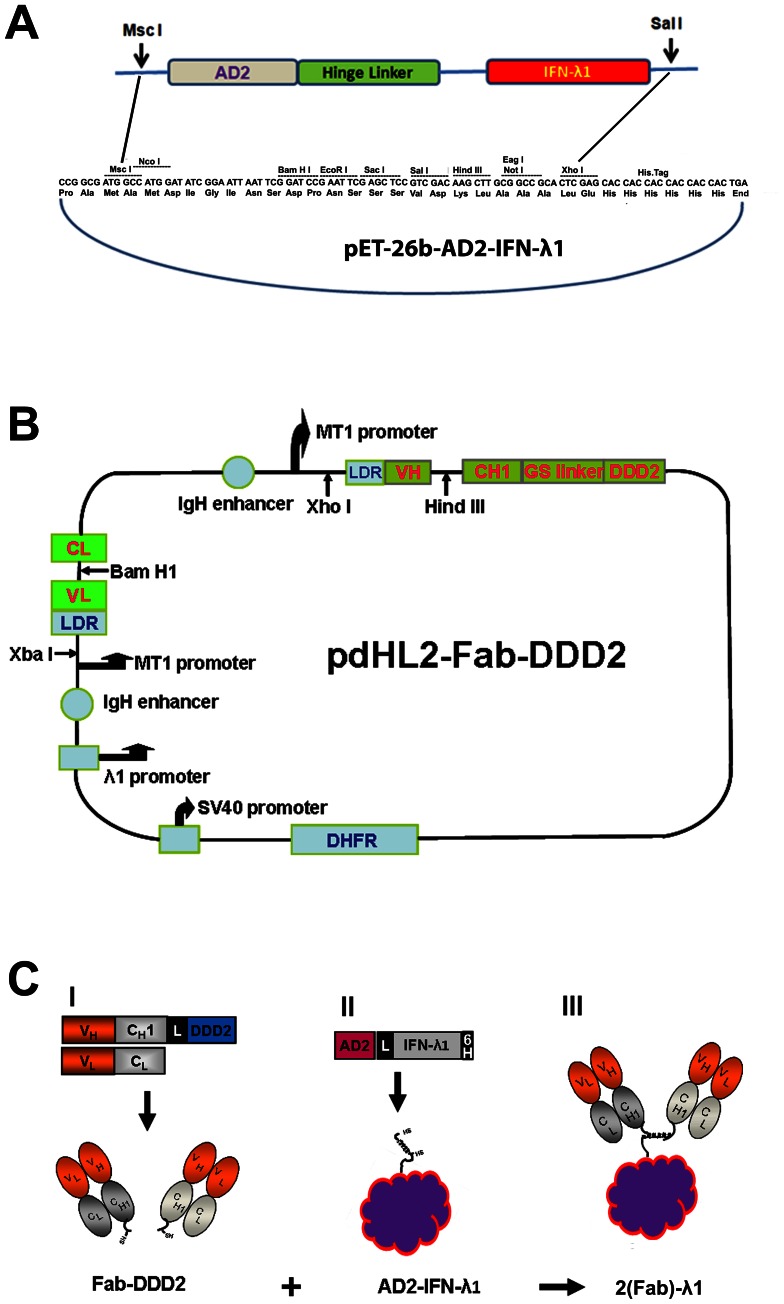
Schematics of AD2-IFN-λ1 and Fab-DDD2 recombinant construction and DNL conjugation. (A) Construction of pET-26b-AD2-IFN-λ1 expression plasmid. The cassette of AD2-hinge linker-IFN-λ1 was synthesized and cloned into the expression vector pET-26b. AD2, an anchoring domain of A kinase anchor protein; hinge linker, a 15 amino acid long flexible linker peptide. (B) Construction of pdHL2-Fab-DDD2 expression plasmids. These constructs were derived from their parental plasmids pdHL2-IgG where the coding sequences for the C_H_1–C_H_3 domains were replaced with a C_H_1-GS-DDD2 cassette. V_H_ and C_H_, variable and constant domains of the heavy chain; V_K_ and C_K_, variable and constant domains of the kappa chain; LDR, leading region; DDD2, the dimerization and docking domain of human PKA-RII protein. (C) DNL conjugation of AD2-IFN-λ1 and Fab-DDD2. The hRS7-Fab-DDD2, hMN15-Fab-DDD2, c225-Fab-DDD2, or hL243-Fab-DDD2 module (I) was docked and locked with AD2- IFN-λ1 module (II) to produce 2(Fab)-λ1, namely (E1)-λ1, (15)-λ1, (c225)-λ1, and (C2)-λ1, respectively (III).

To produce the AD2-IFN-λ1 module used for DNL conjugation, a synthetic cDNA sequence encoding the AD2 peptide, linker peptide (EFPKPSTPPGSSGGAP), and human INF-λ1 with a single point mutation (C171S) were cloned into the Msc I and Xho I restriction sites of the pET-26b vector (Figure 1A). Competent Rosetta-pLysS cells (Novagen, Billerica, MA) were transformed with the expression vector and cultured in shaker flasks at 37°C in Difco 2xYT broth (Becton Dickinson, Franklin Lakes, NJ), supplemented with 100 µg/ml kanamycin sulphate and 34 µg/ml chloramphenicol. When the cell density reached OD_600_ = 1.0, cultures were switched to 30°C and protein expression was induced with 0.5 mM IPTG for 4 h. Cell pellets were frozen, thawed and homogenized in a lysis buffer comprising 2% Triton-X 100, 5 mM MgSO_4_, 10 units/ml benzonase (Novagen), 100 µM 4-(2-aminoethyl) benzenesulfonyl fluoride (Sigma-Aldrich, St. Louis, MO), and 20 mM Tris-HCl, pH 8.0. The insoluble material, containing inclusion bodies, was pelleted by centrifugation, re-homogenized in 1% Triton X-100 in PBS, and re-pelleted. Inclusion bodies were solubilized in 6 M guanidine-HCl, 100 mM Na-phosphate, pH 8.0, and applied to a His-Select affinity column (GE Healthcare, Piscataway, NJ). The denatured protein was eluted in 4 M guanidine-HCl, 100 mM NaH_2_PO_4_, pH 4.5. The eluate was neutralized with 3 M Tris-HCl, pH 8.6, added dithioerythreitol (DTE) to 60 mM, and the resulting solution was held at room temperature overnight. The reduced, denatured protein solution was diluted rapidly into 80-fold volume of 0.5 M arginine, 20 mM oxidized glutathione, 2 mM EDTA; 100 mM Tris, pH 8.0, then dialyzed against 5 L of renaturation buffer (0.5 M arginine, 2 mM oxidized glutathione, 0.6 mM DTE, 2 mM EDTA, 20 mM Tris, pH 8.0) and held for 72 h at 4°C. The solution was then dialyzed against PBS-AG buffer (35.2 mM Na_2_PO_4_.7H_2_O; 0.4 M NaCl; 6.5 mM NaH_2_PO_4_.H_2_O; 150 mM arginine; 150 mM monosodium glutamate, pH 8.0).

Expression vectors for Fab-DDD2, -AD2, or IgG-AD2 modules were engineered from IgG-pdHL2 expression vectors for hRS7, hMN-15, and hL243, as described previously [36] (Figure 1B). The c225-Fab-DDD2 module was constructed using the sequences from DrugBank (Access number: DB00002) as the variable domains of c225. These Fab or IgG modules were produced in myeloma cell culture of SpESFX-10 cells [39] and isolated from culture broths using Kappa or MabSelect affinity chromatography (GE Healthcare, Piscataway, NJ).

### DNL Conjugation

The AD2-IFN-λ1 module was combined with hRS7-Fab-DDD2, hMN15-Fab-DDD2, c225-Fab-DDD2, and hL243-Fab-DDD2 modules to generate (E1)-λ1, (15)-λ1, (c225)-λ1, and (C2)-λ1, respectively, using the DNL method described previously [35], [36]. The DNL products were purified by sequential chromatography on Kappa-select and His-select affinity columns.

### Cell Lines

TE-11 was a gift of Dr. Hiroshi Nakagawa (University of Pennsylvania) [40]. Huh-7 was from the Japanese Collection of Research Bioresources (Osaka, Japan). All other cell lines were from the American Type Culture Collection (ATCC, Manassas, VA). ME-180, TE-11, and A375 were grown in RPMI 1640 media (Life Technologies, Grand Island, NY) with 10% fetal bovine serum (FBS, Thermo Scientific HyClone, Logan, UT). HepG2, Huh-7, and SK-MES-1 were grown in EMEM media (ATCC) with 10% FBS. A549 was grown in F12 media (Life Technologies) with 10% FBS.

### Flow Cytometry

For cell-binding assays, cells were trypsinized briefly, washed, re-suspended in 1% BSA-PBS, and incubated with humanized mAbs (10 µg/ml) or serially diluted IFN-λ1-based agents. Binding was detected with either FITC-labeled goat anti-human IgG or with mouse anti-human IFN-λ1 followed by FITC-labeled goat anti-mouse IgG. All incubations were 45 min at 4°C with 1% BSA-PBS washes between incubations. Binding was measured by flow cytometry using FACS Calibur (BD Biosciences, San Jose, CA).

Changes of MHC-I expression were evaluated by flow cytometry on FACS Calibur following treatment of cells with indicated agents for 3 days. Cells were probed with FITC-labeled mouse anti-human HLA-ABC. FITC-labeled non-specific mouse IgG_1k_ was used as a negative control.

### *In vitro* Proliferation

ME-180, SK-MES-1, and TE-11 were seeded in 96-well plates (1,000 cells/well) and held at 37°C overnight prior to incubation with the indicated agents for 4 days. Viable cells were measured using a CellTiter 96 Cell Proliferation Assay agent (Promega, Madison, WI).

### Antiviral Assays

The anti-HCV activities of rhIFN-λ1, (c225)-λ1, and (C2)-λ1 were measured in Huh-7-Con1 cells (EGFR^+^/HLA-DR^-^), which contain HCV genotype 1b Con1 replicon integrated with a firefly luciferase gene to serve as reporter of viral level [41], [42]. Huh-7-Con1 cells were treated with each of the three agents for 3 days, and the viral replication level was determined by measuring luciferase activity. Meanwhile, the cytotoxicity of these agents was also evaluated on parental Huh-7 cells using CellTiter Glo kit (Promega). The anti-HCV assay was performed by HD Biosciences (China) Co., Ltd (Shanghai, China).

In addition, the antiviral activity of (15)-λ1 was measured by PBL Interferon Source (Piscataway, NJ) on A549 cells (CEACAM6^+^) with EMCV using the cytopathic effect inhibition assay [43]. Included in the assay were hMN-15-Fab-DDD2 as a negative control, rhIFN-λ1 standard (PBL Interferon Source) as a positive control, and (C2)-λ1 as a non-targeting control for structural counterpart.

### SDS-PAGE and Immunoblot Analyses

SDS-PAGE was performed under reducing and non-reducing conditions using 4–20% Tris-Glycine gels (Lonza, Allendale, NJ). To evaluate phosphorylation of STATs, HepG2 cells (5×10^5^) were treated with IFN-λ1 agents for 1 hour prior to lysis with PhosphoSafe™ extraction reagent (Novagen). Cell lysates were resolved on SDS-PAGE, transferred to nitrocellulose membranes, and probed with rabbit antibodies against total STAT1, STAT2, or STAT3 or the phosphotyrosine-specific antibodies pY-STAT1, pY-STAT2, or pY-STAT3. Signals were detected with HRP-goat anti-rabbit antibody. An anti-β-actin antibody was used for assessing β-actin as the loading control.

### RT-PCR Analysis

HepG2 cells were treated with IFN-λ1 agents for 24 h and total RNA was isolated using TRIzol® Reagent (Life Technologies). The mRNA expression of the myxovirus resistance A (MxA) gene was analyzed using SuperScript® III One-Step RT-PCR System (Life Technologies) with primers of forward 5′-AGATCCAGGACCAGCTGAGCCTGT-3′ and reverse 5′-GTGGAACTCGTGTCGGAGTCTGGTA-3′ at conditions: cDNA synthesis-55°C/30 min for one cycle and PCR-94°C/15 sec, 62°C/30 sec, 68°C/30 sec for 25 cycles. Meanwhile, a 452-bp fragment of the glyceraldehyde-3-phosphate dehydrogenase (GAPDH) cDNA was amplified at similar conditions as an internal control [29].

### Pharmacokinetics in Mice

Animal studies were approved by the Center for Molecular Medicine and Immunology Institutional Animal Care and Use Committee, and performed in accordance with the Association for Assessment and Accreditation of Laboratory Animal Care, U.S. Department of Agriculture, and Department of Health and Human Services regulations. Four groups of 8-week-old female athymic nude mice (Taconic, Germantown, NY) were injected subcutaneously with 2.4 nmol of (E1)-λ1 and bled at 6, 16, 24, and 48 hours. Serum concentrations of intact (E1)-λ1 were measured using enzyme-linked immunosorbent assay (ELISA). Pharmacokinetic parameters were calculated using WinNonLin Noncompartmental Analysis Program (Version 5.3, Pharsight Corporation, St. Louis, MO). To corroborate with the results of ELISA, the bioactivity of IFN-λ1 in selective serum samples was also evaluated by *ex vivo* proliferation assay of ME-180 cells, using (E1)-λ1 as a standard.

### ELISA

MaxiSorp 96-well plates (Nunc, Roskilde, Denmark) were coated overnight with goat anti-human F(ab’)2 antibody and blocked with PBS containing 2% bovine serum albumin (BSA) for 1 h. Serum samples for pharmacokinetic study were serially diluted and added to the coated plates. The captured (E1)-λ1 was detected with mouse anti-human IFN-λ1, followed by HRP-conjugated goat anti-mouse IgG Fc antibodies. Pure (E1)-λ1 was included as a standard for quantifying serum samples.

### Statistical Analyses

Statistical significance (*P*<0.05) was determined with F tests for all results using the Prism GraphPad software package (Advanced Graphics Software, Rancho Santa Fe, CA).

## Results

### Generation and Characterizaton of 2(Fab)-λ1

A point mutation (C171S) was introduced into the wild type IFN-λ1 sequence to eliminate the potential interference of the unpaired cysteine residue with the refolding and assembly of the DNL modules. The modified IFN-λ1, with AD2 at its N-terminus, was cloned into pET-26b (Novagen) to produce the expression plasmid of pET-26b-AD2-IFN-λ1 (Figure 1A), which was transformed into *E. coli,* and the AD2-IFN-λ1 module purified from inclusion bodies under denaturing conditions. The four Fab-DDD2 modules, hRS7-Fab-DDD2, hMN-15-Fab-DDD2, c225-Fab-DDD2, and hL243-Fab-DDD2, were prepared using cognate expression plasmids of pdHL2-Fab-DDD2 (Figure 1B), as described previously for hMN-14-Fab-DDD2 [36], and their DNL conjugates with AD2-IFN-λ1 were subsequently generated (Figure 1C), designated (E1)-λ1, (15)-λ1, (c225)-λ1, and (C2)-λ1, respectively. We obtained from standard shaker flask cultures, after refolding, an average yield of 6 mg/L of AD2-IFN-λ1, which was sufficiently pure (Figure S1A) and shown to inhibit the *in vitro* proliferation of ME-180, TE-11, and SK-MES-1 cells as potently as commercial rhIFN-λ1 (Figure S1B). As shown by reducing SDS-PAGE (Figure S1C–F), (E1)-λ1, (15)-λ1, (c225)-λ1, and (C2)-λ1 each consists of only three prominent bands corresponding to AD2-IFN-λ1, Fd-DDD2, and the light chain, indicating a high degree of purity. In addition, the presence of covalently linked 2(Fab)-λ1 was revealed in non-reducing SDS-PAGE (top band in lane 6 of each gel in Figure S1C–F).

### Cell Surface Expression of Antigens

The expression levels of Trop-2, CEACAM6, HLA-DR, and EGFR on the cell surface of seven human cancer cell lines (cervix, ME-180; esophagus, TE-11; lung, A549, SK-MES-1; liver, HepG2, Huh-7; and melanoma-skin, A375) were determined by flow cytometry with hRS7, hMN-15, hL243, and h225 as the probing antibodies. Based on the results (Table 1 and Figure S2), those with a relatively high expression levels of target antigen were considered potential candidates for additional studies, which were ME-180, TE-11, and SK-MES-1 for (E1)-λ1; ME-180, TE-11, HepG2, Huh-7, and A549 for (15)-λ1; and A375 for (C2)-λ1. A high expression of EGFR on Huh-7, ME-180, TE-11, SK-MES-1, and A549 was also noted, suggesting they would be suitable for evaluation of (c225)-λ1.

**Table 1:** Summary of cell binding data (MFI^a^) obtained with parental antibodies.

Cancer	Cell line	Background	CD20	Trop-2	CEACAM6	HLA-DR	EGFR
		GAH-FITC	+hA20	+hRS7	+hMN15	+hL243	+h225
**Cervical**	ME-180	2.9	2.77	979	46.7	2.99	317
**Esophageal**	TE-11	4.22	4.29	433	27.3	4.16	524
**Liver**	HepG2	3.12	3.55	3.1	119	3.14	15
	Huh-7	9.45	9.11	9.82	95.4	9.24	155
**Lung**	A549	3.87	3.94	3.92	162	4.03	61.4
	SK-MES-1	5.96	5.26	48.3	6.32	6.52	190
**Melanoma**	A375	2.75	2.72	2.88	7.01	306	5.22

a Mean fluorescence intensities.

### Enhanced Localization of IFN-λ1 on Cell Surface

**Figure 2:**
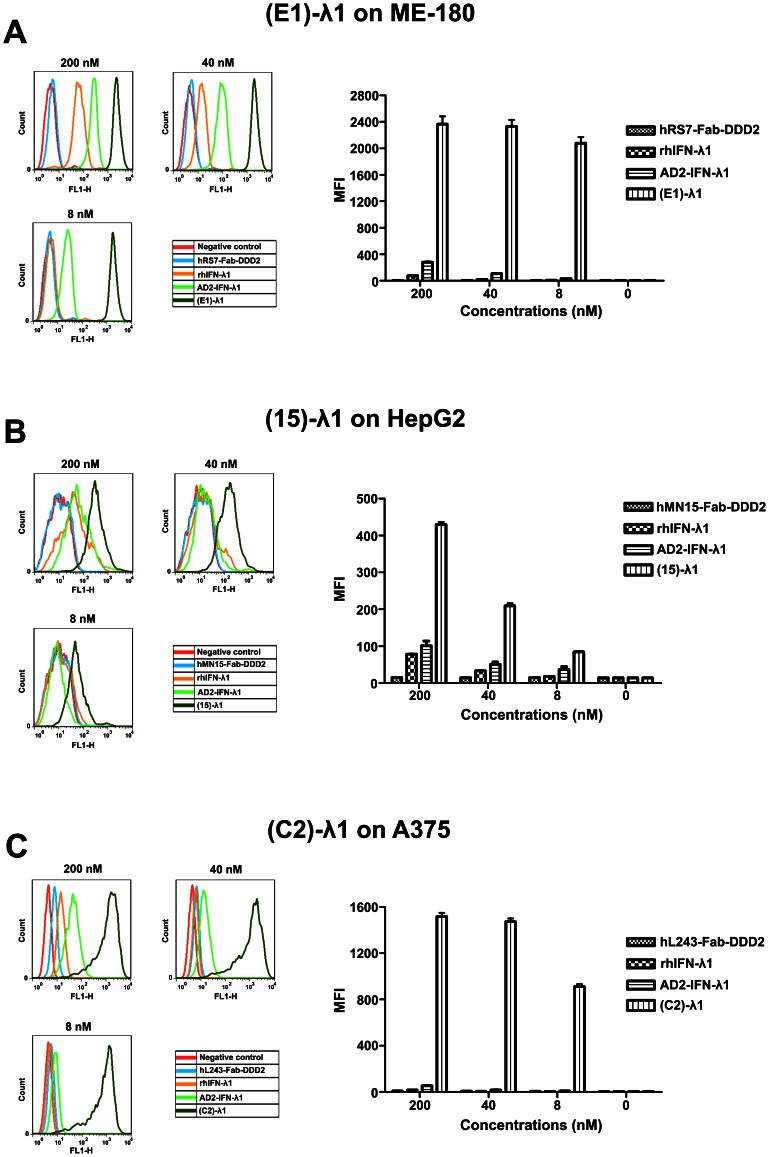
Enhanced binding of 2F (ab)-IFN-λ1 to targeted cells. Cells were incubated at 4°C for 45 min in the presence of 200, 40, 8 nmol/L of indicated agents prior to detection with mouse anti-IFN-λ1 mAb and FITC-labeled goat anti-mouse IgG. MFI, mean fluorescence intensity. N = 3×10,000 cells. Mean ± SD, 95% confidence interval. (A) Enhanced binding of (E1)-λ1 relative to AD2-IFN-λ1 to ME-180 cells. (B) Enhanced binding of (15)-λ1 relative to AD2-IFN-λ1 to HepG2 cells. (C) Enhanced binding of (C2)-λ1 relative to AD2-IFN-λ1 to A375 cells.

Targeting of 2(Fab)-λ1 to antigen-expressing cells markedly increased the amount of the cytokine localized at the cell surface, which could be specifically measured with a mouse anti-human IFN-λ1 followed by FITC-labeled polyclonal goat anti-mouse (GAM) antibodies. As shown in Figure 2A, (E1)-λ1 at 8 nM (the lowest concentration tested) was capable of delivering 100-fold more IFN-λ1 to the surface of ME-180 (Trop-2^+^) cells than the untargeted AD2-IFN-λ1. Likewise, (15)-λ1 and (C2)-λ1 at 8 nM achieved 15- and 500-fold higher mean fluorescence intensities (MFIs) than AD2-IFN-λ1 in HepG2 (CEACAM6^+^) and A375 (HLA-DR^+^) cells, respectively (Figure 2B, C).

### Effects on *in vitro* Growth

**Figure 3:**
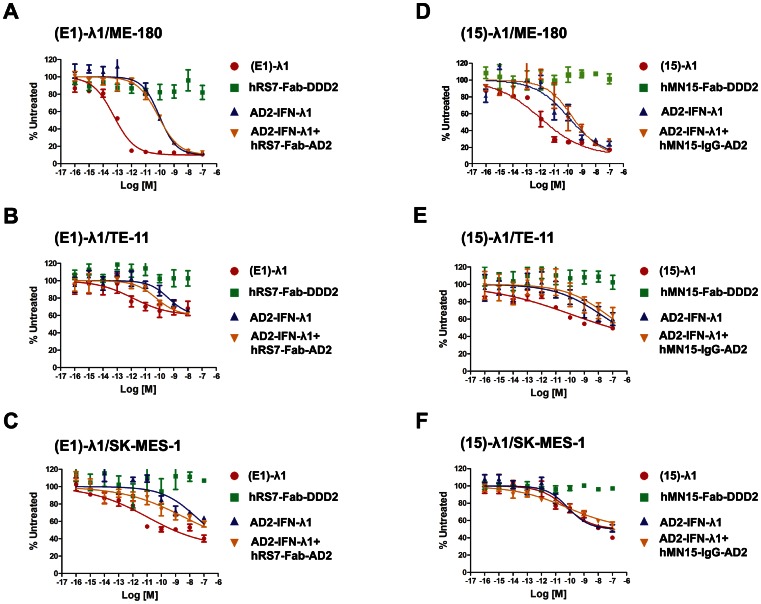
*In vitro* growth suppression and cytotoxicity. Indicated cell lines were cultured for 4 days in the presence of increasing concentrations of (E1)-λ1 (A-C) or (15)-λ1 (D-F), and the relative cell viabilities were measured with MTS. The percentage of the signal obtained from untreated cells was plotted versus the log of the molar concentration. Dose–response curves and EC_50_ values were generated using Prism software. Each study also included parallel evaluation of constitutive modules (AD2-IFN-λ1, hRS7-Fab-DDD2, hMN15-Fab-DDD2) tested alone, or AD2-IFN-λ1 and control modules (hRS7-Fab-AD2, hMN-15-IgG-AD2) tested in combination.

The potency of (E1)-λ1 to inhibit *in vitro* growth of target cells was compared with its two constitutive modules (AD2-IFN-λ1 and hRS7-Fab-DDD2) alone, or with a combination of AD2-IFN-λ1 and hRS7-Fab-AD2 (as a surrogate of hRS7-Fab-DDD2), in ME-180 (Figure 3A), TE-11 (Figure 3B), and SK-MES-1 (Figure 3C) cells. Similar studies were performed for (15)-λ1 in ME-180 (Figure 3D), TE-11 (Figure 3E) and SK-MES-1 (Figure 3F). Based on the dose-response curves obtained (Figures 3A–F and S1B), the following observations can be made: (*i*) in each of the three cell lines tested, neither hRS7-Fab-DDD2 nor hMN-15-Fab-DDD2 showed any inhibitory activity up to 100 nM, whereas AD2-IFN-λ1 was as effective as rhIFN-λ1(Figure S1B); (*ii*) the addition of hRS7-Fab-AD2 (Figures 3A–C) or hMN-15-IgG-AD2 (Figures 3D–F) to AD2-IFN-λ had no effect on the inhibitory potency of AD2-IFN-λ1; (*iii*) (E1)-λ1 inhibited ME-180 with an EC_50_<0.1 pM, which was over 1000-fold more potent than AD2-IFN-λ1 (EC_50_ ∼100 pM) (Figure 3A); (E1)-λ1 also exhibited higher anti-proliferative activity than AD2-IFN-λ1 against TE-11 (Figure 3B) and SK-MES-1 (Figure 3C) cells; (*iv*) (15)-λ1 had 100-fold higher potency compared to AD2-IFN-λ1 in ME-180 (Figure 3D), and was more effective under the maximal dose (100 nM) tested in TE-11 (Figure 3E), both of which express moderate levels of CEACAM6; in contrast, (15)-λ1 was no more potent than AD2-IFN-λ1 in CEACAM6-negative SK-MES-1 cells (Figure 3F); (*v*) (E1)-λ1 (EC_50_ = 0.1 pM; Figure 3A) was 10-fold more active than (15)-λ1 (EC_50_ = 1 pM, Figure 3D) on ME-180, which expresses higher Trop-2 than CEACAM6.

### Antiviral Activity

**Figure 4:**
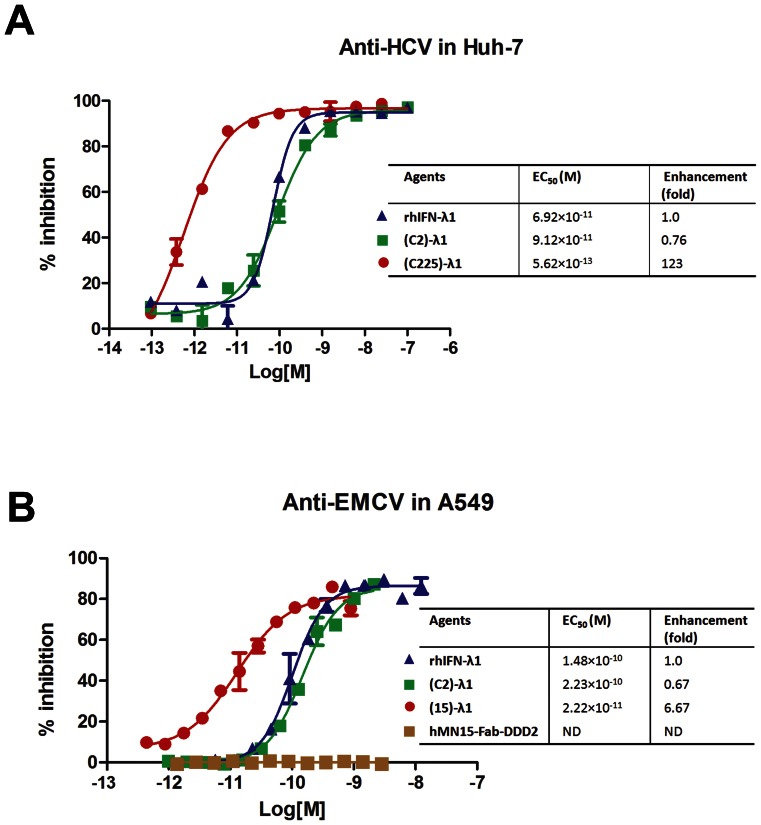
Antiviral effects. (A) Enhanced anti-HCV potency of (c225)-λ1 in Huh-7 cells. A Huh-7 stable cell line with HCV genotype 1b Con1 replicon expressing firefly luciferase was treated with indicated concentrations of (c225)-λ1, (C2)-λ1, or rhIFN-λ1 agents. After 3 days, luciferase activity was measured and antiviral effects were determined by percent activity reduction relative to untreated cells. Data were analyzed by Graph Pad Prism using a sigmoidal fit (variable slope). Samples were run twice independently in duplicate. (B) Enhanced anti-EMCV potency of (15)-λ1 in A549 cells. A549 cells were incubated with serial dilutions of (15)-λ1, (C2)-λ1, rhIFN-λ1, or hMN15-Fab-DDD2 before being challenged with EMCV. A visual cytopathic effect (CPE) determination was performed, and the data were analyzed by GraphPad Prism using a sigmoidal fit (variable slope). Samples were run twice independently in duplicate. Mean ± SD, 95% confidence interval.

The antiviral activity of selective 2(Fab)-λ1 constructs was measured against HCV and EMCV in Huh-7 and A549 cells, respectively. In EGFR-expressing Huh-7 cells, (c225)-λ1 (EC_50_ = 0.56 pM) was 163- and 123-fold more potent at inhibiting HCV replication (Figure 4A) than the non-targeting (C2)-λ1 (EC_50_ = 91.2 pM) and commercial rhIFN-λ1 (EC_50_ = 69.2 pM), respectively. In CEACAM6-expressing A549 cells, (15)-λ1 compared to the non-targeting (C2)-λ1 and rhIFN-λ1 showed 10- and 6-fold higher anti-EMCV activity, respectively (Figure 4B). Notably, (C2)-λ1 retains 65 to 75% antiviral activity of rhIFN-λ1. These results indicate that cell surface targeting of IFN-λ1 can effectively enhance its antiviral activity.

### Cell Signaling

**Figure 5:**
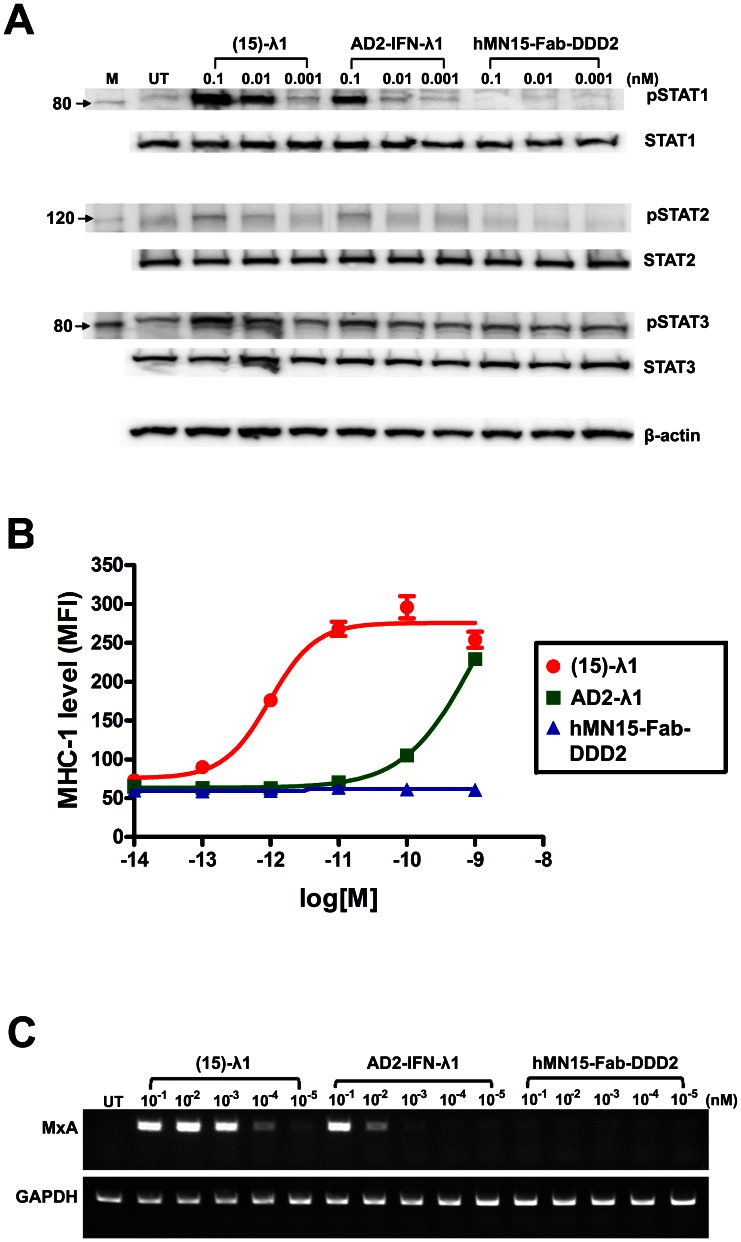
IFN-λ1-induced cell signaling. (A) Immunoblot analysis of STAT phosphorylation. HepG2 cells were treated with indicated test articles for 1 h, and phosphorylated STAT1, 2, and 3 were measured with loading 30 µg of total protein/lane. Total STATs and β-actin were probed to verify equal loading. (B) Flow cytometric analysis of MHC class I antigen expression. HepG2 cells were cultured for three days in the presence of increasing concentrations of (15)-λ1, AD2-IFN-λ1, or 15-Fab-DDD2 and then stained with FITC labeled mouse anti-human HLA-ABC or isotopic-matched control Ab. MFI, mean fluorescence intensity. N = 10,000 cells. Data were analyzed by FlowJo software (Tree Star, Ashland, OR). (C) RT-PCR analysis of MxA mRNA expression. HepG2 cells were treated with IFN-λ1 agents for 24 h, and the mRNA expression of MxA gene was analyzed in 100 ng of total RNA/sample. GAPDH cDNA was amplified as an internal control for cDNA amounts. Data are representative of two independent experiments.

The activation of STATs upon binding (15)-λ1 to HepG2 cells was evaluated. As shown in Figure 5A, (15)-λ1 at 10 pM induced a notable phosphorylation of STAT1, which was observed with AD2-IFN-λ1 at 100 pM, but not 10 pM. Under the same conditions, hMN15-Fab-DDD2 had no effect. An increase of phosphorylated STAT3 above the basal levels was also discernable with (15)-λ1 at 10 pM or AD2-IFN-λ1 at 100 pM. For STAT2, only a slight elevation of phosphorylation could be detected. The enhanced STAT phosphorylation by (E1)-λ1 or (C2)-λ1 over AD2-IFN-λ1 was also observed in ME-180 or A375 cell line (Figure S3).

The ability of (15)-λ1 to up-regulate the cell surface expression of MHC class I antigens (MHC-I) also was investigated in HepG2 cells by flow cytometry (Figure 5B). Whereas treatment of HepG2 cells with hMN-15-Fab-DDD2 up to 1 nM for three days gave no change in the surface levels of MHC-1 (MFI ∼50), a more than 3-fold increase in MFI (∼170) was observed in cells treated with (15)-λ1 as low as 1 pM. The expression of MHC-1 reached a maximal level (MFI ∼270) with (15)-λ1 at 100 pM. Although AD2-IFN-λ1 was capable of up-regulating MHC-1, it required a higher concentration (1 nM) to be as effective.

We also examined the capability of (15)-λ1 to induce the expression of the myxovirus resistance A (*MxA*) gene, a known marker for IFN bioactivity, and the results of RT-PCR are shown in Figure 5C. In untreated or hMN-15-Fab-DDD2-treated HepG2 cells, the mRNAs of *MxA* were undetectable by RT-PCR. The induction of the *MxA* gene was evident in cells treated with 0.1 pM of (15)-λ1 or 10 pM of AD2-IFN-λ1, thus further attesting to the advantage of (15)-λ1 over AD2-IFN-λ1 in particular, and 2(Fab)-λ1 over IFN-λ1 in general.

### Pharmacokinetics in Mice

**Figure 6:**
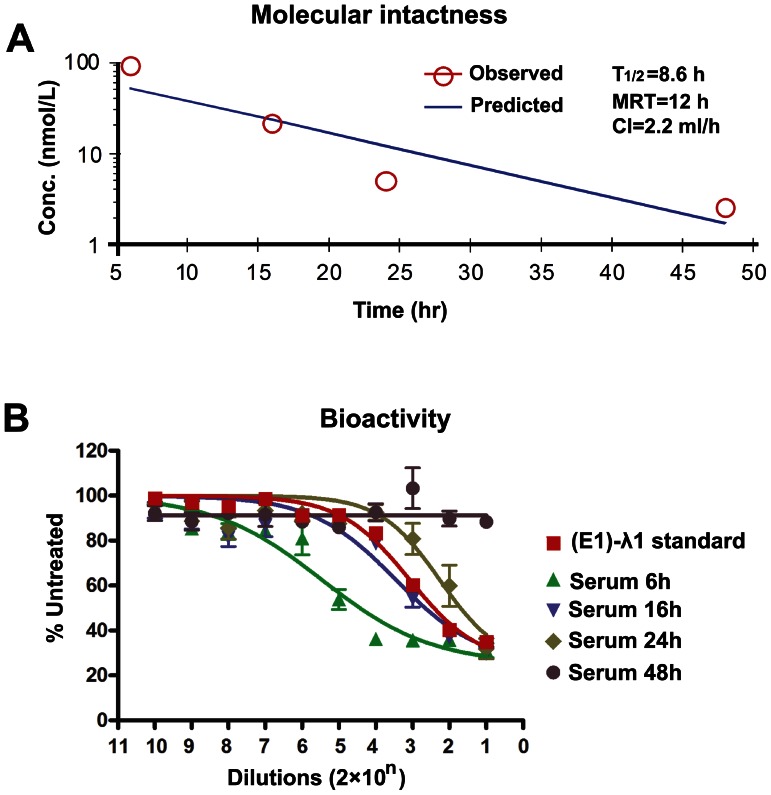
Pharmacokinetics in mice. (A) Mean serum concentration of intact molecules versus time in mice after a single dose (2.4 nmol/animal) of (E1)-λ1. The pharmacokinetic parameters are derived from noncompartmental analysis of ELISA data. T_1/2_, half life; MRT, mean residence time; Cl, clearance. N = 3 animals/time point. Mean ± SD, 95% confidence interval. (B) Specific bioactivity of serum samples in the *ex vivo* assay of ME-180 cell growth. Serums were diluted at 1∶20 as the initial test concentration, and then serially diluted at 1∶10. (E1)-λ1 was used as a standard control with test concentrations from 1 nM to 10^−9^ nM. Cells were treated for 4 days, and viable cells were measured with MTS. Dose–response curves were generated using Prism software. N = 3 animals/time point. Mean ± SD, 95% confidence interval.

After a single dose (2.4 nmol) of subcutaneous administration, the mean serum concentration of intact (E1)-λ1 reached a high level by 6 h and fell below the limit of ELISA detection by 48 h (Figure 6A). The pharmacokinetic (PK) parameters derived from noncompartmental analysis demonstrate a mean residence time of 12 h with a T_1/2_ of 8.6 h, and clearance of 2.2 ml/h. When the concentrations of (E1)-λ1 in the serum sample were also measured by its inhibitory activity on ME-180 cells using the MTS assay, the results were largely consistent (Figure 6B).

As shown in our previous study [37], recombinant IFN-α has a very rapid rate of clearance in mice, showing a mean residence time of only 0.7 h. Thus, 2(Fab)-λ1 demonstrates a significantly improved PK that is comparable to PEG-IFN-α [37] and PEG-IFN-λ [44].

## Discussion

The potential of IFN-λ as a therapeutic alternative to IFN-α is being explored with PEG-IFN-λ1, which shows an improved safety profile over PEG-IFN-α-2a in clinical studies [4], [33]. However, rates of some serious adverse events, including dose-limiting hepatotoxicity, are similar for patients treated with PEG-IFN-λ1 and PEG-IFN-α-2a, and even more frequent in patients treated with the highest dose of PEG-IFN-λ1 [34]. On the other hand, IFN-λs were less effective than type I IFNs against certain cancers [26] or viruses [45]. We postulated that linking IFN-λs to an antibody specific for an abundantly expressed surface antigen could enhance its localization at the target cells, resulting in greater potency and, hopefully, less toxicity to non-target cells. Accordingly, we developed four prototypes of IFN-λ-based immunocytokines, each comprising IFN-λ1 conjugated site-specifically to a stabilized dimer of Fab, and demonstrated their superiority compared to the unconjugated parental modules alone or in combination. The improved effects were shown in both antitumor and antiviral assays, and are consistent with enhanced cell-surface binding and signal transduction, which are enabled by the constitutive antibody.

In the antitumor studies, we found ME-180 to be the most sensitive cell line to IFN-λ1, with over 80% maximum inhibition and a 20-fold lower EC_50_ than other cell lines reported in the literature (Table S1) [25]–[29]. Moreover, the abundant expression of Trop-2 on ME-180 renders these tumor cells highly responsive to (E1)-λ1, with growth inhibition detectable at 1 fM and an EC_50_ (<0.1 pM) 1000-fold lower than AD2-IFN-λ1 (EC_50_ ∼100 pM). Similar enhancements of inhibitory potency also were observed in TE-11, SK-MES-1, and other cancer cell lines. Because IFN-λs also induce innate and adaptive immune responses, which were not evaluated here, and in view of recent studies showing that the constitutive expression of IFN-λ in several murine cancer cell lines, including B16 melanoma, BNL hepatoma, and MCA205 fibrosarcoma, despite the lack of *in vitro* antiproliferative activity, markedly suppressed tumor growth and metastasis in syngeneic mouse models by recruiting immune cells and related cytokines [30], [46], [47], we posit that targeted delivery of IFN-λ will resemble constitutive expression of IFN-λ in cancer cells, resulting in a local immune response and enhanced cytotoxicity in the immunotherapy of cancer [48].

In the antiviral studies, the specific targeting of (c225)-λ1 to EGFR-positive Huh-7 cells hosting HCV genotype 1b Con1 replicon exhibited 123- and 163-fold enhancement of antiviral potency over non-targeting rhIFN-λ1 and (C2)-λ1, respectively. In another assay, the targeting of (15)-λ1 to CEACAM6-positive A549 cells challenged with EMCV exhibited 6- and 10-fold improvement of antiviral protection over the non-targeting rhIFN-λ1 and (C2)-λ1, respectively. The difference in potency between (c225)-λ1 and (15)-λ1 is likely due to the distinct sensitivity of their targeted cell/virus systems to IFN-λ1. In a previous study [17], rhIFN-λ1 was 10-fold less potent than rhIFN-α in the Huh-7/HCV system, and 210-fold less potent in A549/EMCV [26]. Although the enhancement of (15)-λ1-induced antiviral activity is not as high in target cells, compared to (c225)-λ1, it is still a significant finding, considering that pegylated IFNs only retain about 30% or less activity of unpegylated IFNs [49], and the specific activity of AD2-IFN-λ1 was similar to rhIFN-λ1. Thus, 2(Fab)-λ1 may allow a lower dose with the same or less frequent dosing schedule than the PEG-IFN-λ1 used in current clinical studies.

As a therapeutic agent, recombinant IFN is limited by its very rapid rate of clearance. Based on our previous study, recombinant IFN-α-2b, PEG-IFN-α-2a, and PEG-IFN-α-2b exhibited a half-life of 0.7, 14.9, and 9.3 h, respectively [37]. Thus, a comparable half-life of (E1)-λ1 in mice (8.6 h) to the two regulatory approved PEG-IFN-αs is encouraging, and merits further *in vivo* experiments in murine and non-human primate models.

## Supporting Information

Figure S1 **Generation and characterization of 2(Fab)-λ1.** (A) SDS-PAGE analysis of refolded AD2-IFN-λ1 module. M, Mr standard; lanes 1 and 3, BSA; lanes 2 and 4, AD2-IFN-λ1; R, reducing; NR, nonreducing. (B) Bioactivity comparison between AD2-IFN-λ1 and commercial rhIFN-λ1. ME-180, TE-11, and SK-MES-1 cells were grown in the presence of increasing concentrations of AD2-IFN-λ1 or rhIFN-λ1 and the relative viable cell densities were measured with MTS. Dose–response curves were generated using Prism software. (C-F) SDS-PAGE analysis of purified (E1)-λ1, (15)-λ1, (C225)-λ1, or (C2)-λ1 and their constituents under reducing (R) and non-reducing (NR) conditions. Reducing condition resolved three bands representing polypeptides for Fab-DDD2-heavy chain, kappa light chain, and AD2-IFN-λ1, and non-reducing condition resolved a major high-relative mobility band representing the covalent DNL structure. Lanes: M, Mr standards; 1 and 4, AD2-IFN-λ1; 2 and 5, hRS7-, hMN15-, C225-, or hL243-Fab-DDD2; 3 and 6, (E1)-λ1, (15)-λ1, (c225)-λ1, or (C2)-λ1.
(TIF)

Figure S2 **Flow cytometric analysis of cell surface antigens.** Cells were incubated with 10 µg/ml humanized parental antibodies on ice for 45 min, probed with FITC labeled goat anti-human IgG (FITC-GAH), and then measured by flow cytometry. No primary antibody and humanized anti-CD20 IgG (hA20) were used as background and negative controls respectively. Data were analyzed by FlowJo software and shown in Table 1.
(TIF)

Figure S3 **Immunoblot analysis of STAT phosphorylation.** Cells were treated with indicated test articles for 1 h, and phosphorylated STAT-1, -2, and -3 were measured with loading 30 µg of total protein/lane. Total STATs and β-actin were probed to verify equal loading. (A) ME-180 cells; (B) A375 cells.
(TIF)

Table S1 ***In vitro*** **proliferation sensitivity of cancer cell lines to rhIFN-λ1.**
(DOCX)
