# Supplementary figures and images for: Interferon-λ1 Linked to a Stabilized Dimer of Fab Potently Enhances both Antitumor and Antiviral Activities in Targeted Cells
Source: PLoS One. 2013 May 16;8(5):e63940. doi: 10.1371/journal.pone.0063940 (PMC3655979; doi:10.1371/journal.pone.0063940)

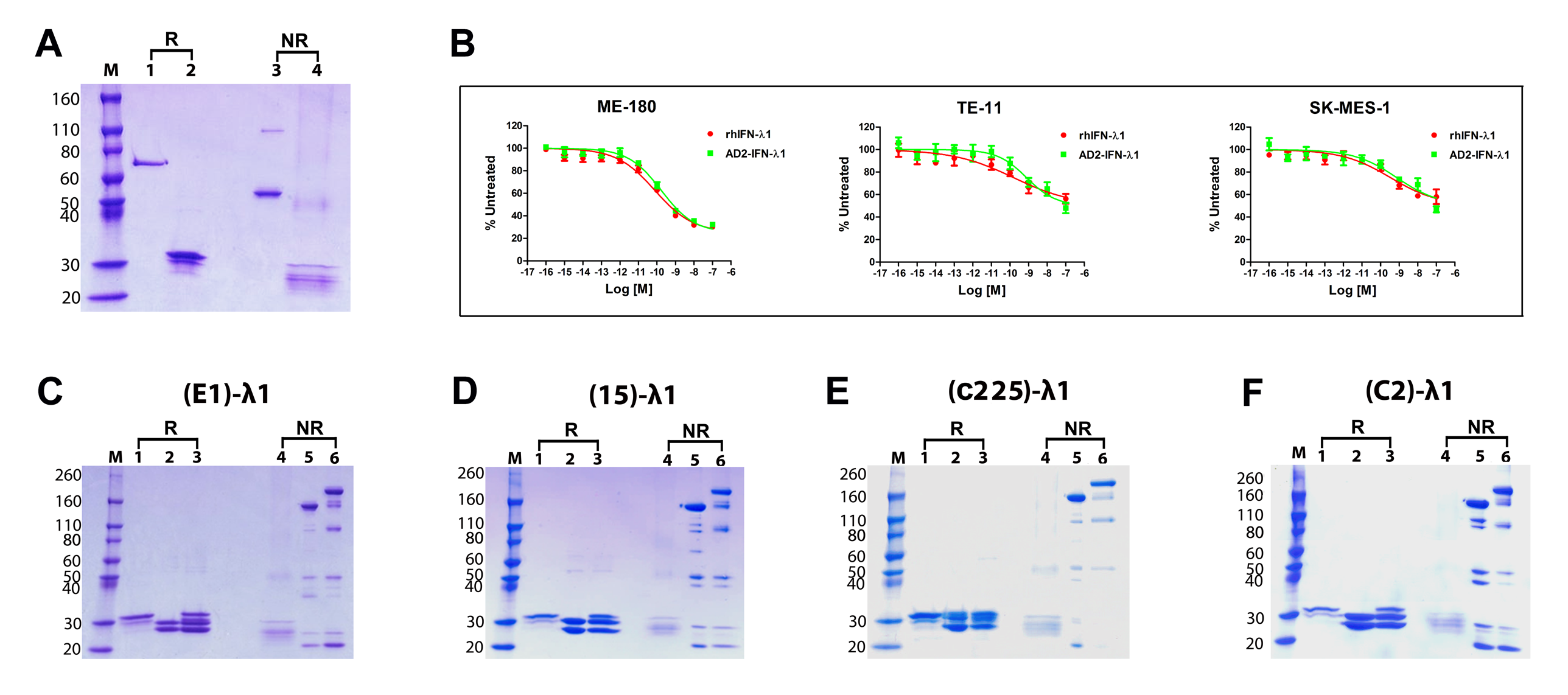

Supplement: Figure S1 — Generation and characterization of 2(Fab)-λ1. (A) SDS-PAGE analysis of refolded AD2-IFN-λ1 module. M, Mr standard; lanes 1 and 3, BSA; lanes 2 and 4, AD2-IFN-λ1; R, reducing; NR, nonreducing. (B) Bioactivity comparison between AD2-IFN-λ1 and commercial rhIFN-λ1. ME-180, TE-11, and SK-MES-1 cells were grown in the presence of increasing concentrations of AD2-IFN-λ1 or rhIFN-λ1 and the relative viable cell densities were measured with MTS. Dose–response curves were generated using Prism software. (C-F) SDS-PAGE analysis of purified (E1)-λ1, (15)-λ1, (C225)-λ1, or (C2)-λ1 and their constituents under reducing (R) and non-reducing (NR) conditions. Reducing condition resolved three bands representing polypeptides for Fab-DDD2-heavy chain, kappa light chain, and AD2-IFN-λ1, and non-reducing condition resolved a major high-relative mobility band representing the covalent DNL structure. Lanes: M, Mr standards; 1 and 4, AD2-IFN-λ1; 2 and 5, hRS7-, hMN15-, C225-, or hL243-Fab-DDD2; 3 and 6, (E1)-λ1, (15)-λ1, (c225)-λ1, or (C2)-λ1. (TIF) [file pone.0063940.s001.tif]

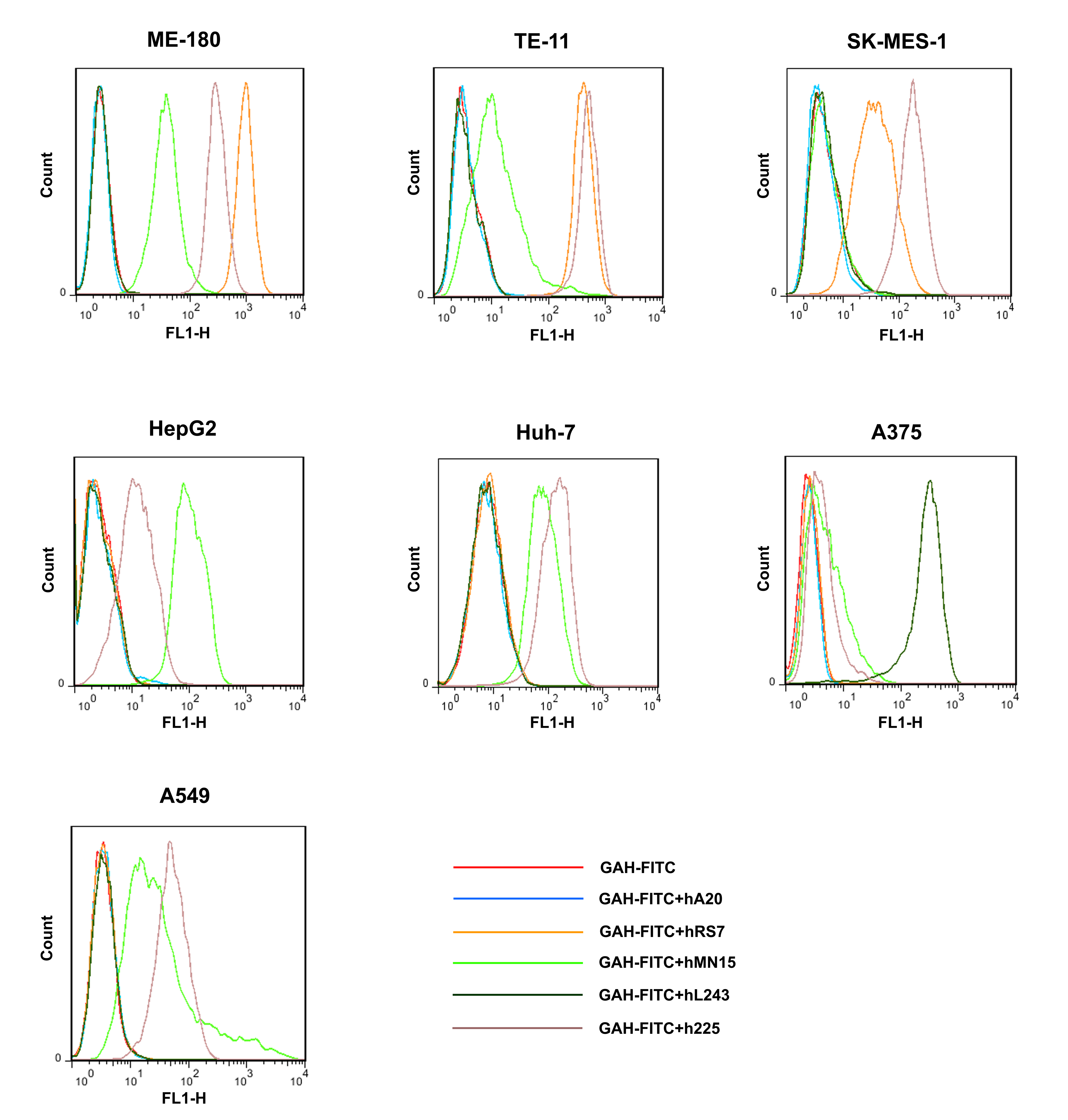

Supplement: Figure S2 — Flow cytometric analysis of cell surface antigens. Cells were incubated with 10 µg/ml humanized parental antibodies on ice for 45 min, probed with FITC labeled goat anti-human IgG (FITC-GAH), and then measured by flow cytometry. No primary antibody and humanized anti-CD20 IgG (hA20) were used as background and negative controls respectively. Data were analyzed by FlowJo software and shown in Table 1. (TIF) [file pone.0063940.s002.tif]

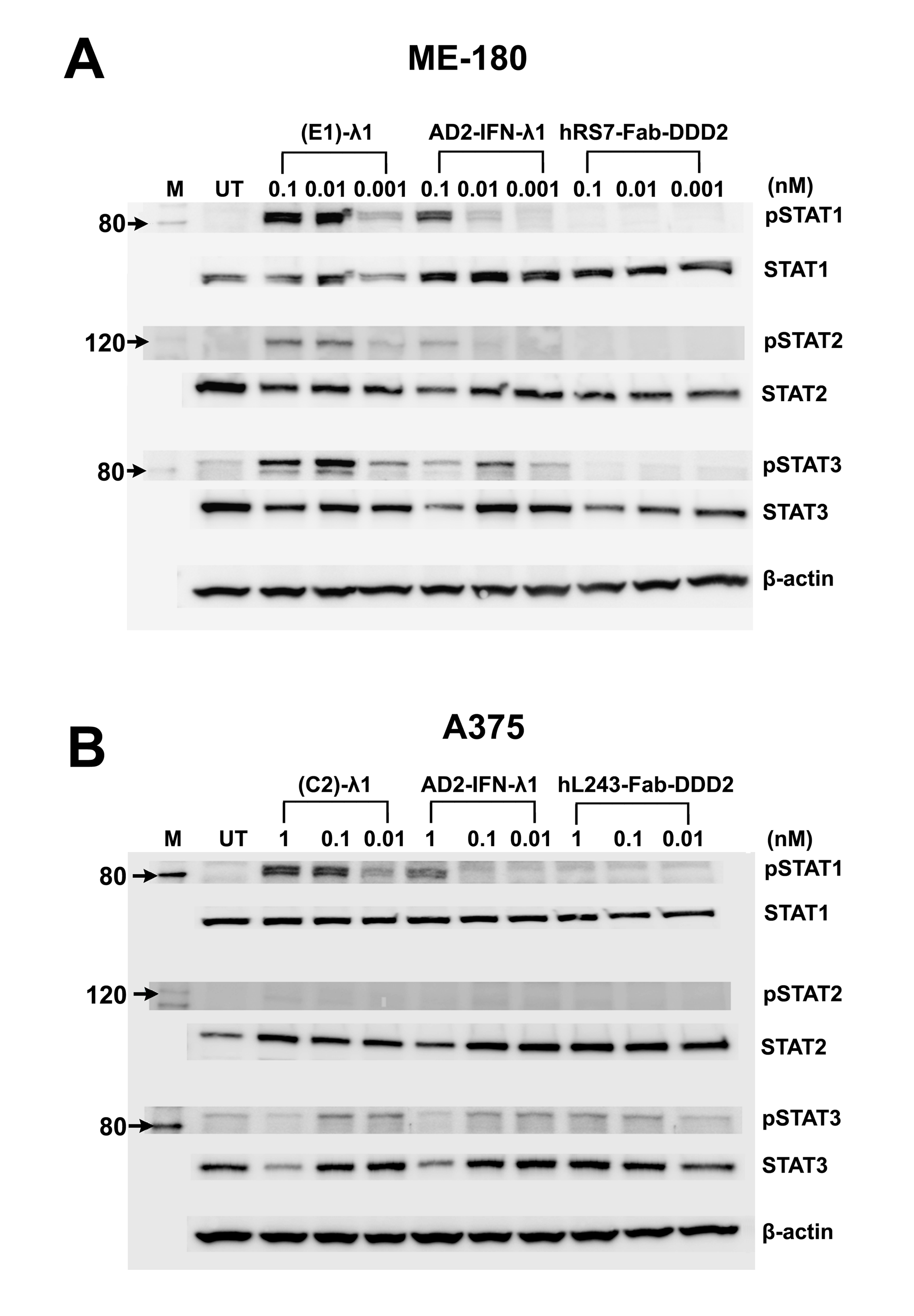

Supplement: Figure S3 — Immunoblot analysis of STAT phosphorylation. Cells were treated with indicated test articles for 1 h, and phosphorylated STAT-1, -2, and -3 were measured with loading 30 µg of total protein/lane. Total STATs and β-actin were probed to verify equal loading. (A) ME-180 cells; (B) A375 cells. (TIF) [file pone.0063940.s003.tif]
