# Supplementary material for: Interferon-λ1 Linked to a Stabilized Dimer of Fab Potently Enhances both Antitumor and Antiviral Activities in Targeted Cells
Source: PLoS One. 2013 May 16;8(5):e63940. doi: 10.1371/journal.pone.0063940 (PMC3655979; doi:10.1371/journal.pone.0063940)
Supplement: Table S1 — In vitro proliferation sensitivity of cancer cell lines to rhIFN-λ1. (DOCX) [file pone.0063940.s004.docx]

**Table S1. *In vitro* proliferation sensitivity of cancer cell lines to rhIFN-λ1**

| **Cancer** | **Cell line** | ***MI** | †**EC_MI_ (ng/mL)** | **EC_50_ (ng/mL)** | **Reference** |
| --- | --- | --- | --- | --- | --- |
| **Cervical** | ME-180 | 80% | 20 | 2 | This study |
| **Esophageal** | TE-11 | 75% | 1000 | 50 | [29] |
| **Keratinocyte** | HaCaT | 45% | 100 | 50 | [27] |
| **Neuroendocrine** | BON1 | 30% | >10 | ‡NA | [25] |
| **Glioblastoma** | LN319 | 75% | 400 | 120 | [26] |
| **Melanoma** | F01 | 15% | 100 | NA | [28] |

*MI= Maximal inhibition

†EC_MI_ = Effective concentration to achieve maximal inhibition

‡NA=not available
